# Influence of Omega-3 Fatty Acid Status on the Way Rats Adapt to Chronic Restraint Stress

**DOI:** 10.1371/journal.pone.0042142

**Published:** 2012-07-30

**Authors:** Marie Hennebelle, Laure Balasse, Alizée Latour, Gaelle Champeil-Potokar, Stéphanie Denis, Monique Lavialle, Pascale Gisquet-Verrier, Isabelle Denis, Sylvie Vancassel

**Affiliations:** 1 INRA, Unité de Nutrition et Régulation Lipidiques des Fonctions Cérébrales, NuRéLiCe, UR909, Domaine de Vilvert, Jouy en Josas, France; 2 CNRS, Centre de Neurosciences Paris-Sud, Univ Paris-Sud UMR 8195, Orsay, France; 3 Université Paris-Sud, UMR 8195, Orsay, France; State University of Rio de Janeiro, Biomedical Center, Institute of Biology, Brazil

## Abstract

Omega-3 fatty acids are important for several neuronal and cognitive functions. Altered omega-3 fatty acid status has been implicated in reduced resistance to stress and mood disorders. We therefore evaluated the effects of repeated restraint stress (6 h/day for 21 days) on adult rats fed omega-3 deficient, control or omega-3 enriched diets from conception. We measured body weight, plasma corticosterone and hippocampus glucocorticoid receptors and correlated these data with emotional and depression-like behaviour assessed by their open-field (OF) activity, anxiety in the elevated-plus maze (EPM), the sucrose preference test and the startle response. We also determined their plasma and brain membrane lipid profiles by gas chromatography. Repeated restraint stress caused rats fed a control diet to lose weight. Their plasma corticosterone increased and they showed moderate behavioural changes, with increases only in grooming (OF test) and entries into the open arms (EPM). Rats fed the omega-3 enriched diet had a lower stress-induced weight loss and plasma corticosterone peak, and reduced grooming. Rats chronically lacking omega-3 fatty acid exhibited an increased startle response, a stress-induced decrease in locomotor activity and exaggerated grooming. The brain omega-3 fatty acids increased as the dietary omega-3 fatty acids increased; diets containing preformed long-chain omega-3 fatty acid were better than diets containing the precursor alpha-linolenic acid. However, the restraint stress reduced the amounts of omega-3 incorporated. These data showed that the response to chronic restraint stress was modulated by the omega-3 fatty acid supply, a dietary deficiency was deleterious while enrichment protecting against stress.

## Introduction

One disturbing feature of western diets is the growing imbalance between omega-6 and omega-3 fatty acids (FA) that may restrict the supply of long-chain omega-3 FA (LC-FA) to the tissues and lead to a mild omega-3 FA deficiency [1], [2]. LC-FA, especially docosahexaenoic acid (DHA; 22:6n-3) and arachidonic acid (AA; 20:4n-6), are fundamental components of the membrane phospholipids of neural cells. DHA and AA can be provided directly from the diet or by metabolism of their precursors, alpha-linolenic acid (ALA; 18:3n-3) and linoleic acid (LA; 18:2n-6). Many studies have investigated the effect of an inadequate omega-3 FA nutritional intake on brain function and cognitive disorders because DHA is more abundant in the brain than in most other tissues [3]. Incorporation into the brain occurs mainly during the perinatal period [4], but the dietary supply also plays an important role in adults by compensating for the daily loss of brain DHA [5]–[7]. Actually, the dietary intake of omega-3 LC-FAs is depends almost exclusively on fish consumption. But most of the population eat very little fish, which contributes to an inadequate peripheral and cerebral DHA status [1], [2].

Epidemiological data have shown a negative correlation between peripheral markers of omega-3 FA consumption and psychological distress, anxiety and depression [6], [8]–[11]. And meta-analyses have shown that the omega-3 FA intake helps protect against mood disorders, with significant benefits in unipolar and bipolar depression [12], [13].

Studies on animals fed a low omega-3 FA diet have clearly shown behavioural disorders in deficient animals [14], [15]. In particular, a dietary-induced loss of brain DHA influences the reaction and sensitivity of individuals to stress and mood disorders [16], [17]. We used the paradigm of early maternal separation as social stressor to show that adult rats that had been separated and kept on a chronic dietary omega-3 FA deficiency were more impulsive than stressed rats fed a balanced omega-3 FA diet. They reacted more to novelty, were anxious and fearful and exhibited changes in their reward responses [18], [19], particularly when coping with stressful situations. The corollary has also been demonstrated: diets with adequate or enriched in omega-3 FA have positive effects on anxiety and depressive-like symptoms in rodents [20], [21].

These data suggest that a combination of stress and a lack of omega-3 FA could be a powerful stimulus altering cognition and mood, while dietary supplement of omega-3 FA could help optimize brain resistance to stress-induced responses and damage.

The early physiological response to stress is the activation of the hypothalamo-pituitary-adrenal (HPA) axis leading to elevated circulating concentrations of glucocorticoids. Brain glucocorticoid receptors (GR) appear to be very sensitive to the glucocorticoid concentration and the high capacity of this receptor is connected with its role in the negative feedback inhibition of the HPA, so helping the system to return to its basal level [22]. The concentration of GR is particularly high in the hippocampus, which correlates with its role in modulating stress responsiveness through negative feedback, and this high concentration also makes the hippocampus a major target for the various and maladaptive effects of glucocorticoids that lead to related cognitive disorders [23]–[26]. Chronic/repeated exposure to stressful situations can influence the development and severity of several disorders, such as depression and depression-like behaviour, through dysregulation of the HPA [27].

This study was designed to characterise the combined effect of the dietary omega-3 FA supply and repeated exposure to stress on the physiology and behaviour of rats. We used an ALA deficient diet to produce male rats with brain phospholipid DHA levels 50% lower than those fed a control diet containing adequate ALA and an omega-3 LC-FA enriched diet to produce rats with brain phospholipid DHA concentrations 10% higher than these controls, as assessed by analysis of the lipid profiles of the plasma and brain membranes. Middle-aged rats (6 month-old) raised under each of these different diets were subjected to chronic restraint stress (6 h/d) for 21 days, adapted from that described by Borcel et al, [28]. This mimics the psychogenic stress that is frequently observed in humans after repeated exposure to stress. The rationale for stressing middle-aged rats at was based on evidence that this period is a one when rats are particularly vulnerable to continuous stress [29]. The 21 days of daily restraint stress cause long-lasting changes in the morphology of hippocampus cells [30], accompanied by alterations in anxiety, activity and emotional behaviour [31]–[34]. We evaluated the differences in the body weights, plasma corticosterone concentrations and the abundance of GR in the hippocampus of the rats on each diet. These were correlated with emotional and depression-like behaviour assessed by their open-field (OF) activity, their anxiety in the elevated-plus maze (EPM), the sucrose preference test and the startle response. These variables were selected because similar responses can be measured in humans, providing valid human parallels and making them useful for understanding sensitivity to stress and its relationship to nutritional status.

## Materials and Methods

### Experimental designs and tissue preparation

#### Ethics statement

All experiments were performed in accordance with the European Communities Council Directive (86/609/EEC) and the French Department of Agriculture (licence n° 67–88 to E.C.). The research was conducted under the authorization n° 78–29 from the “Direction Départementale des Services Vétérinaires des Yvelines”. All efforts were made to minimize the number, pain and discomfort to the rats.

#### Animals and diet

Rats were raised under conditions of controlled temperature (22±1°C), humidity (50±10%) and light cycles (7 a.m.–7 p.m.) and were given water *ad libitum*. Two weeks before mating, female Wistar rats were placed on the control diet (CON) containing a mixture of high-oleic sunflower and rapeseed oils (rich in ALA), or on the omega-3 FA deficient diet (DEF) containing only sunflower oil, or on the omega-3 LC-FA enriched diet (ENR) containing sunflower and tuna oils (**Tables 1** **and** **2**). All experiments were done on the first generation of adult male rats born to mothers in each dietary group. Four litters were used for each dietary group and only males were kept. Then, on postnatal day 1, groups of 10–12 males were randomly assigned to a foster dam with the corresponding diet. This random distribution of the males among the dams was carried out to redistribute possible effects of genetic and prenatal factors and to obtain similar litter size. From weaning, pairs of rats with the same diet were housed in the same cage and fed the same diet as their mothers. Each rat was given 25 g food/day from weaning to 3 months of age and then 20 g/day until they were 6-months old to avoid the development of obesity commonly observed when rats were fed *ad libitum*. Rats were weighed every two weeks from weaning to 6 months and once a week during the stress procedure.

**Table 1:** FA composition of the experimental diets (% weight of total FA)^1^.

	CON Diet	DEF Diet	ENR Diet
**Saturated**	**7.3**	**7.8**	**14.9**
**Σ Monounsaturated**	**65.6**	**69.8**	**58.3**
18: 2 n-6	22.0	22.4	15.6
20: 4 n-6	-	-	0.6
**Σ omega-6 FA**	**22.0**	**22.4**	**16.3**
18: 3 n-3	5.2	0.1	0.2
20: 5 n-3	-	-	2.3
22: 6 n-3	-	-	7.4
**Σ omega-3 FA**	**5.2**	**0.1**	**10.6**
	*mg/100 g diet*		
18: 2 n-6	1307	1370	955
20: 4 n-6	-	-	37
18: 3 n-3	309	6	12
20: 5 n-3	-	-	141
22: 6 n-3	-	-	453

1 FA, fatty acids; 20:4n-6, arachidonic acid (AA); 20:5n-3, eicosapentaenoic acid (EPA); 22:6n-3, docosahexaenoic acid (DHA); DEF, Deficient diet; CON, Control diet; ENR, Enriched diet.

**Table 2:** Macro and micronutrients composition of the experimental diets^1^.

	CON Diet	DEF Diet	ENR Diet
	*(g/100 g diet)*		
Casein (vitamin free)	22	22	22
DL-methionine	0.2	0.2	0.2
Cellulose	2	2	2
Mineral mix^2^	4	4	4
Vitamin mix^3^	1	1	1
Corn starch	42.6	43.2	42.7
Sucrose	21.3	21.6	21.4
Fats			
African peanut oil	3.8	6.0	4.6
Rapeseed oil	2.2	-	-
Tuna oil	-	-	2.2

1 Both diets provided 16.5 MJ/kg; lipids provided 13.5% of total calories. Oils were kindly supplied by Lesieur-Alimentaire (Coudekerque; France).

2 (g/100 g): CaHPO_4_-2H_2_O: 38; K_2_HPO_4_: 24; CaCO_3_: 18; NaCl: 6.9; MgO: 2; MgSO_4_-7H_2_O : 9 ; FeSO_4_-7H_2_O : 0.86 ; ZnSO_4_-H_2_O : 0.5 ; MnSO_4_-H_2_O : 0.5 ; CuSO_4_-5H_2_O : 0.1 ; NaF : 0.08 ; CrK(SO_4_)_2_-12H_2_O : 0.05 ; (NH_4_)_6_Mo_2_O_24_-4H_2_O : 0.002 ; KI : 0.004 ; CoCO_3_ : 0.002 ; Na_2_SeO_3_ : 0.002.

3 Composition of vitamin supplement triturated in dextrose (mg/kg of vitamin mixture) : retinyl acetate (UI), 500000; cholecalciferol (UI), 250000; acetate dl-alpha-tocopherol (UI), 5000; menadione (UI), 100; thiamine HCl (UI), 1000; riboflavine, 1000; nicotinic acid, 4500; D-calcium panthotenate, 3000; pyridoxine HCl, 1000; inositol, 5000; D-biotin, 20; folic acid, 200; cyanocobalamin, 1.35; L-ascorbic acid, 10000; paraamino-benzoic acid, 5000; choline chlorhydrate, 75000.

DEF, Deficient diet; CON, Control diet; ENR, Enriched diet.

#### Repeated restraint stress procedure

Half of the 6-month old male rats from each dietary group were subjected to a validated chronic stress procedure (Buyntsky and Mostosky, 2009) based on a restraint stress procedure adapted from Veena et al, [32]. Rats were placed in wire mesh restrainers for 6 h a day (fom 10 am to 16 pm) for 21 days (5 days a week) over a period of 4 weeks. Unstressed rats were handled weekly. The unstressed and stressed animals were housed in different rooms. The 6 experimental groups were: unstressed DEF, stressed DEF, unstressed CON, stressed CON, unstressed ENR and stressed ENR.

One week after the end of the restraint stress procedure, part of the rats (n = 16 per group) was subjected to different behavioral tests, while another part (n = 10 per group) was killed by decapitation and their brains dissected out on ice. The frontal cortex was isolated and its FA content measured. Similarly, the CA1 region of the hippocampus was isolated and it content of corticoid hormone receptors measured.

### Determination of plasma corticosterone

Blood samples were taken on day 0 (basal), and on days 1, 7, 14 and 21 of the restraint stress procedure, 30 min after the beginning of restraint stress. Blood was collected from the tail vein into tubes containing heparin (Microvette CB, Sarstedt, Marnay, France) and centrifuged at 3000 g for 10 min at 4°C. The resulting plasma was stored at −80°C. Plasma corticosterone was determined by radioimmunoassay, using the Immuchem™ Double Antibody Corticosterone 125I RIA kit (MP Biomedicals, Orangeburg, NY, USA) and the procedure recommended by the manufacturer. All samples were run in duplicate. Values were read off from the standard curve and results are expressed as ng/mL plasma. Intraassay and interassay variations were less than 10% and less than 15%, respectively.

### Behavioural measurements

The tests were done in the following sequence: OF, EPM, sucrose preference test and startle response test.

#### Video tracking system

The ANY-maze™ flexible video tracking system (Stoelting Co, Wood Dale, USA) was used to automate testing in the OF and EPM experiments. The camera was placed above the apparatus, linked to the monitor in an adjoining room, and behaviors were scored live.

#### Open Field

Rats were first tested in an OF to determine their exploratory locomotor activity and their emotional reactivity/anxiety state by recording self-grooming, a behaviour that plays a major role in coping with stress [35] and increases with high stress [36], [37]. The OF, constructed of white painted wood, was a 100×100 cm square, divided in 25 (20×20 cm) squares, and surrounded by a 50 cm high wall. The OF test was conducted in a brightly lit room (140–200 lux). Each rat was placed in a corner of the OF for 5 min. Its horizontal locomotor activity, expressed as the number of squares crossed, was monitored using the Any-maze video tracking system. The number of rearings and the time spent in grooming were registered by the experimenter. At the end of each test, the rat was returned to its home cage and the OF was cleaned with 60% aqueous ethanol. The locomotor exploratory activity was defined as the sum of crossings and rearings, and the time spent in self-grooming was measured.

#### Elevated Plus Maze

The EPM test was performed on next day to determine each rat's basal anxiety and was further use to approximate the motor activity [38]. The EPM apparatus, constructed of black PVC, was 50 cm above the floor. Four arms (50 cm long and 11 cm wide) with 50-cm high walls, two open and two closed, were arranged in a cross with two arms facing each other and intersecting at a central neutral zone (10×10 cm). The EPM test was conducted in a dimly lit room (30–40 lux). Each animal was placed in the center of the EPM, facing a closed arm, and followed for 5 min of free exploration with the Any-maze Video Tracking System. An entry was recorded if half of the body crossed the entrance of an open or closed arm (this index has been selected because the numbers of full body entrance were too low). The time spent in each arm and the number of entries into them was measured. Rats were returned to their home cages at the end of the 5 min test and the EPM was cleaned with 60% aqueous ethanol.

#### Sucrose preference test

This test is used to investigate the anhedonic state, a core symptom of depressive-like behavior that can be generated by chronic stress in rats [39]. On the day after the EPM test, each rat was placed in a cage equipped with two water bottles for 24 h for habituation, and they remained in the same cage throughout the test. On the next day, the rat was deprived of water for 20 h [40]. Then the rat was offered two pre-weighed bottles, one containing tap water and the other a 1% sucrose solution, for 12 hours. The bottles were removed and weighed to determine the amounts of water and sucrose drunk. The percent sucrose preference is expressed by: sucrose intake×100/water+ sucrose intake.

#### Startle response test

The startle response test began the day after the measure of sucrose preference; it was used to investigate fear and anxiety [41]. Two startle chambers (Columbus Instruments, Ohio, USA) were used to measure the startle response. Each chamber consisted of a Plexiglas box (16×28×18 cm) resting on a platform placed inside a sound-attenuated, ventilated chamber that produced 68 dB of continuous broadband background noise. A high-frequency loudspeaker inside each chamber delivered the acoustic stimuli produced by a tone-generator. Sound levels within each test chamber were measured routinely using a sound level meter to ensure consistent presentation. The peak amplitude of the whole body startle to an acoustic pulse was measured (in grams) within the first 100 milliseconds from the onset of stimulus presentation, by a Responder-X system (Columbus Instruments, Columbus, Ohio, USA). Rats were placed inside the apparatus and the startle session started with a 3 min acclimatization period of 68 dB background noise, which continued throughout the session. The rats were then given a series of five 1 dB stimuli (20 ms duration) separated by 20 s, to define the basal weight for each rat. They then were given a series of ten 120 dB stimuli (30 ms duration), separated by intervals of 15 to 25 s. The startle response was obtained by subtracting the basal weight (in grams) from the peak amplitude.

### Assay of glucocorticoid hormone receptors

#### GR protein analysis by Western blotting

Samples of CA1 were homogenised in RIPA buffer containing antiproteases (Sigma) and their protein content assayed using the DC Assay method. Aliquots (30 microg) of protein were loaded onto 4–15% Tris-HCl- polyacrilamide gels (BioRad), separated by electrophoresis (40 mA for 1 h), and transferred to polyvinylidene fluoride membranes (45 µm, Immobilon-P, Millipore) by electrotransfer for 2 h at 30 V at 4°C. The membrane free binding sites were blocked by incubation for 2 h at room temperature in Tris-buffered saline/Tween-20 (TBS/T) (25 mM Tris base, 137 mM NaCl, 2.7 mM KCl and 0.1% Tween-20, pH 7.6) containing 5% dried milk. The membranes were then incubated with the primary rabbit polyclonal anti-GR antibody, (1∶500, Santa Cruz: Biotechnologies, California, USA) diluted in TBS/T containing 5% dried milk. They were then washed several times with TBST and incubated with the secondary antibody (1∶30000; peroxidase conjugated donkey anti-rabbit IgG, Jackson Immunoresearch Laboratories) diluted in TBST containing 5% dried milk. Immunoreactive bands were detected by enhanced chemiluminescence (ECL+ reagent, Amersham, France) and quantified using a camera connected to image analysis software (AIDA, Raytest, Courbevoie, France). The GAPDH concentrations were measured to ensure equal loading. Results are expressed as the GR receptor immunoreactivity/GAPDH ratio, and as a percent of the values obtained for unstressed control rat samples in the same gel.

#### Assays of GR mRNA by real-time quantitative RT-PCR

Samples of CA1 were frozen at −20°C in RNA-later buffer. Total RNA was extracted from each sample using a MirVana miRNA isolation kit according to the manufacturer's instructions (Ambion, France) and the quantity of RNA measured using a NanoDrop 1000 spectrophotometer (Thermo Scientific, France). The quality of the RNA was determined on a bioanalyser 2100 (Agilent Technology, France) that calculates the 28S:18S ratio and the RNA integrity number (RIN). All samples used had a RIN>8 and an A_260_/A_280_>1.8. Total RNA for each sample was reverse transcribed with the High capacity cDNA Reverse Transcription (RT) kit (Applied Biosystems, Foster City, CA) in a final volume of 100 µl contained 1 x RT buffer, 1 x RT random primers, 4 mM dNTP Mix and 250 U Multiscribe Reverse Transcriptase.

Real-time quantitative PCR was performed using the ABI Prism 7900 Sequence Detection Systems (Applied Biosystems). cDNA samples were diluted with nuclease-free water (1∶4), and amplified in a 20 µl reaction volume with 10 µl 2× TaqMan Universal Master Mix, plus 1 µl 20× Assays-on-Demand (Rn00561369_m1 for GR and Rn01775763_g1 for GAPDH). The thermal cycling conditions were: initial denaturation at 95°C for 10 min plus 40 cycles at 95°C for 15 s and at 60°C for 1 min. All experiments were performed in triplicate for each data point. Data were collected with Applied Biosystems SDS version 2.1 software and analysed with RQ Manager Version 1.2 software (Applied Biosystems). GAPDH was used to normalize gene expression levels for further analysis of q-PCR experiments using the delta-delta Ct method [42].

### Lipid analysis

#### FA profile of phospholipid classes in the frontal cortex

The membrane phospholipid composition in the frontal cortex was considered to reflect changes in the total brain [18]. Total lipids were extracted by a modification of the Folch method [43]. Phosphatidylethanolamine (PE), the phospholipid class with the highest DHA content, was separated from total lipids by solid-phase extraction on a silica column [44]. The FAs were transmethylated by incubation with boron trifluoride at 90°C for 20 min (Fluka, Sokolab) and then analyzed by gas chromatography [45]. Results are expressed as percentage weight (g/100 g of total FA (TFA; wt %)).

#### Plasma FA composition

Blood was collected onto heparin at the time of sacrifice, centrifuged and the plasma was stored at −80°C. Total lipids were extracted from the plasma with chloroform/methanol 2/1 using the method of Folch [43] and then transmethylated with boron trifluoride at 90°C for 60 min. The total lipid fractions were then separated by solid phase liquid chromatography and FA methyl esters were analyzed by gas chromatography [45]. Results are expressed as percentage weight (TFA; wt %).

### Statistical Analysis

Data are shown as means ± SEM. Statistical analyses were performed using StatView (version5, SAS Institute Inc., Cary, North Carolina, USA). The main effects and interaction of the two independent variables, diet and stress, were evaluated using two-way ANOVA. For body weight and plasma corticosterone data, repeated-measures ANOVA with factors of diet and day was conducted. Significance was set at *p<0.05*. Then Multiple Comparison Procedures (MCP) based on Bonferroni correction were run, with p values considered significant when <0.01.

## Results

### Body weight

All the pups had identical weights at birth (6.5±0.2 g), regardless of the litter or the mother's dietary regimen, suggesting that diet had no effect on embryonic development. ENR rats gained weight thereafter more rapidly than the rats in the two other groups (Diet x Day interaction: F(28,1161) = 8.211, p<0.001; p<0.05 for ENR rats vs. CON and DEF, respectively). The ENR rats weighed 10% more (633.2±9.9 g) than the CON (580.2±7.7 g, p<0.001) and DEF rats (574.2±7.1 g, p<0.001) at age 6 months, prior to starting the restraint stress procedure.

**Figure 1:**
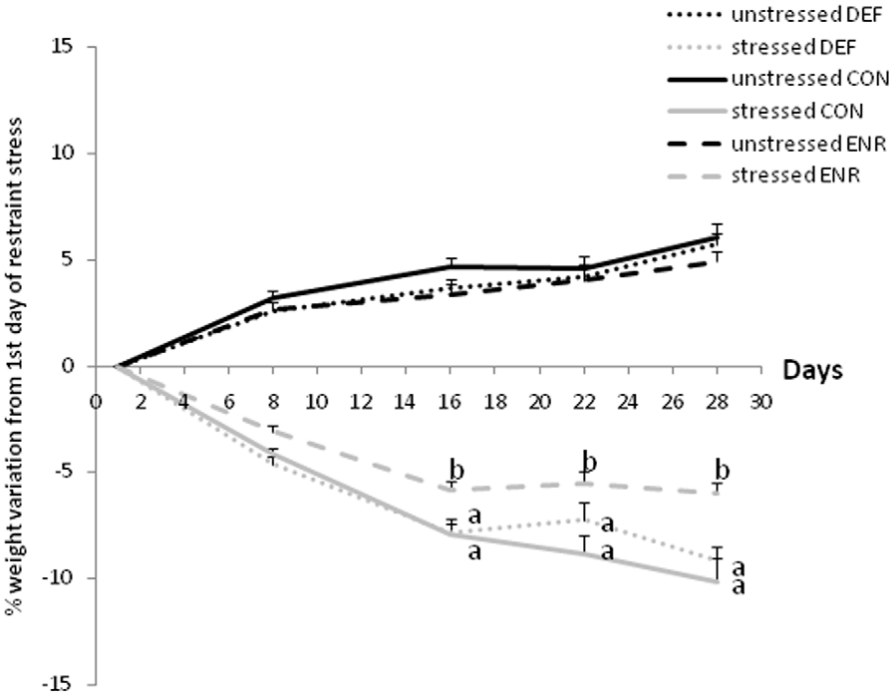
Changes in body weight during the restraint stress. Values are means ± SEM (% from the first day of restraint stress; n = 16 per group). Data from the stressed rats with different superscript letters are significantly different between dietary groups on that measurement day (ANOVA followed by MCP, *p<0.01*). DEF, Deficient diet; CON, Control diet; ENR, Enriched diet.

The rats in all dietary groups lost weight during the 21 days of restraint stress (Day: F(4,139) = 89.250, p<0.05) while all the unstressed groups continued to grow (+6% as a mean; p<0.05) (**Figure 1**). However, the ENR rats lost less weight than rats in the other two groups (Diet x Day interaction: F(8,139) = 2.921, p<0.01; p<0.05 for ENR rats vs. CON and DEF). At the beginning of the behavioural tests, all groups of rats exhibited body weights that did not significantly differ any longer: unstressed CON: 645.5±10.6 g, stressed CON: 553.5±10.7 g; unstressed DEF: 602.8±9.7 g, stressed DEF: 573.0±8.3 g; unstressed ENR: 636.0±9.1 g, stressed ENR: 588.8±8.4 g 636.0 (diet factor: F(2,18) = 0.126, p = 0.883; stress factor: F(1,18) = 3.947, p = 0.062; no interaction).

### Plasma corticosterone and glucocorticoid receptors

**Figure 2:**
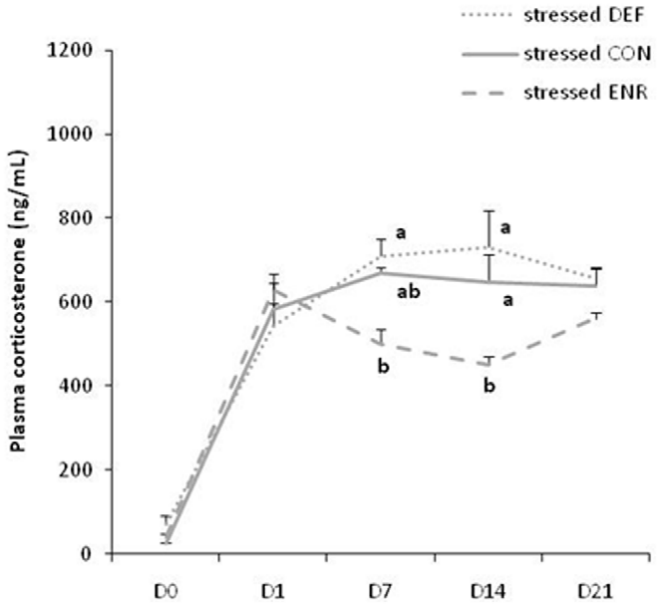
Plasma corticosterone during the 21 days of restraint stress. Plasma corticosterone was measured before starting the restraint stress (D0) and on days 1, 7, 14 and 21 30 min after beginning the stress. Values are means ± SEM (ng/mL; n = 9 per group). Data with different superscript letters are significantly different between dietary groups on that measurement day (ANOVA followed by MCP, *p<0.01*). DEF, Deficient diet; CON, Control diet; ENR, Enriched diet.

The basal plasma corticosterone concentration measured one day before the beginning of the restraint stress procedure (D0) was not affected by the diet (mean: 45.1±7.2 ng/mL for all 3 groups). The plasma corticosterone monitored for one session (6 hours) of restraint stress indicated that it reached a maximum 30 min after the start (T_30_) of the restraint in all groups of rats (data not shown). The plasma corticosterone concentrations of all dietary groups were elevated in samples taken at T_30_ on days 1, 7, 14 and 21 of the restraint stress procedure as compared to basal value (**Figure 2**). But the increase in corticosterone was less in the stressed ENR rats on days 7 and 14 than in the rats in the other dietary groups (Diet: F(2,56) = 4.777, p<0.05; Diet x Day: F(6,56) = 3.656, p<0.05; D7: p = 0.006 and p = 0.033 for ENR rats vs. DEF and CON, respectively; D14: p<0.001 and p = 0.002 for ENR rats vs. DEF and CON, respectively). We also determined corticosterone level in non stressed rats 28 days later and once again there was no diet effect on (data not shown).

The amounts of GR mRNA and protein in the CA1 area were determined one week after the end of the restraint stress procedure. The relative abundances of GR mRNA in the unstressed rats was not affected by the diet (F(2,29)<1), and they were not significantly modified by stress (F(1,29)<1). Similarly, the amounts of GR protein were not influenced by stress: the amounts of GR protein normalized to GAPDH and expressed as percent of the unstressed CON rats were: 149.7±33.3 for stressed CON; 114.4±32.0 for unstressed DEF and 143.5±41.9 for stressed DEF; 111.2±11.2 for unstressed ENR and 119.1±23.6 for stressed ENR.

### Behavioural measurements

#### Open Field

**Figure 3:**
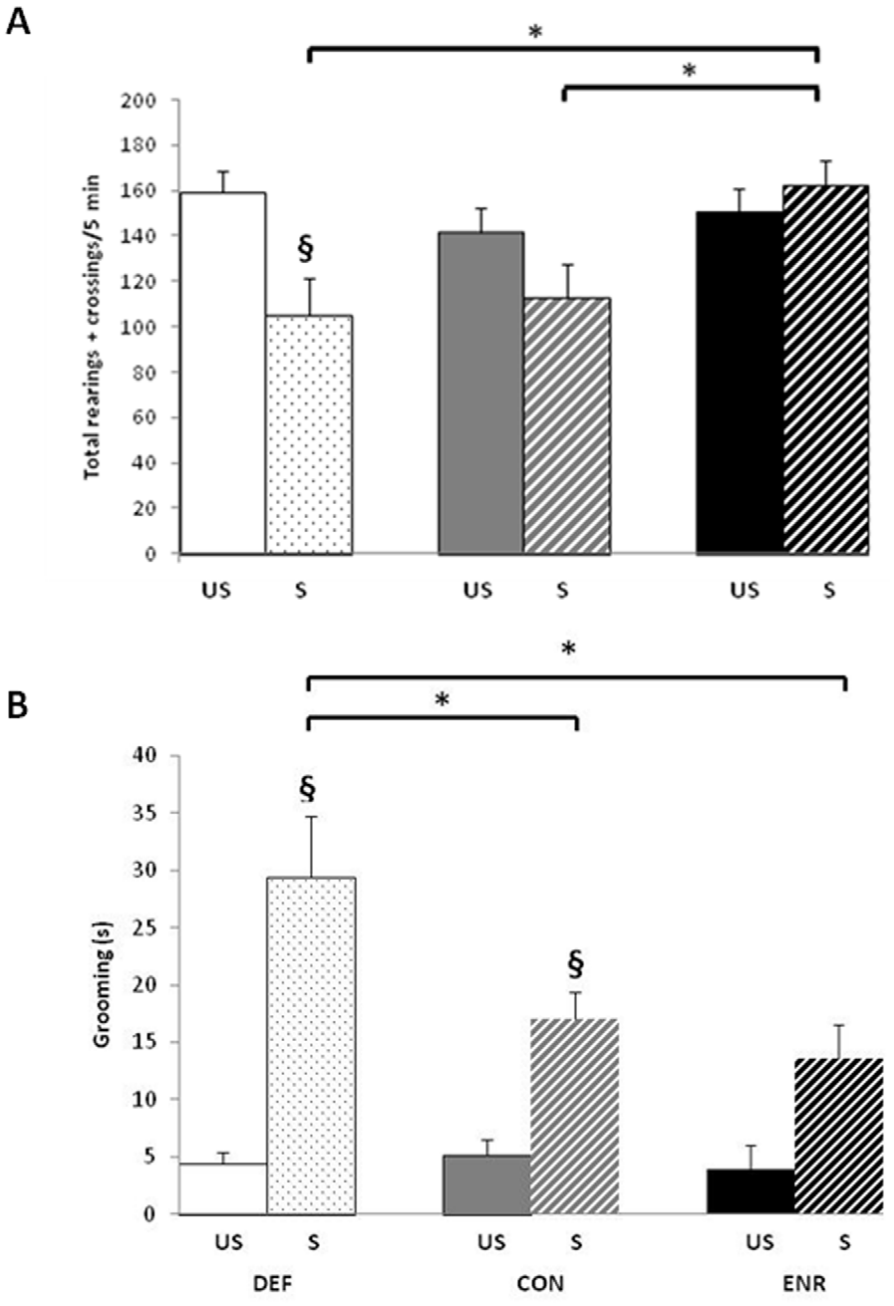
Performance in the Open Field (OF) test. **A**. Motor activity, defined as the sum of crossings and rearings. **B**. Time of grooming (s). Values are means ± SEM (n = 16 per group). **§** Significantly different from unstressed rats in the same dietary group; * significantly different between stressed groups (ANOVA followed by MCP, *p<0.01*). US, unstressed; S, stressed; DEF, Deficient diet; CON, Control diet; ENR, Enriched diet.

Two-way ANOVA on motor activity, expressed as the sum of rearings and crossings during the 5 min test, indicated a significant effect of diet (F(2,66) = )3.214, p<0.05), of stress (F(1,66) = 5.613, p<0.05) and a significant diet x stress interaction (F(2,66) = 3.635, p<0.05) (**Figure 3A**). MCP revealed that in the DEF groups, stressed rats had a motor activity score significant lower than unstressed rats (p = 0.0028). This effect of stress was greatly attenuated in CON rats, resulting in no significant difference between the stressed and unstressed rats (p = 0.10); and there was no difference at all between the stressed and unstressed ENR rats. Hence, the ENR stressed rats had higher activity scores than the stressed CON (p = 0.0058) and the stressed DEF rats (p = 0.0018).

The stressed rats in CON and DEF groups spent significantly more time grooming during the OF than did the unstressed rats (Stress: F(1,61) = 40.575, p<0.0001; DEF: p<0.0001; CON: p = 0.0056; ENR: p = 0.0309) (**Figure 3B**). Furthermore, this effect of stress was significantly greater in DEF rats, who groomed more than the stressed CON (p = 0.0003) and ENR rats (p = 0.0042) (Diet: F(2,61) = 3.962, p<0.05; Diet x Stress: F(2,61) = 3.918, p<0.05).

#### Elevated Plus Maze

**Figure 4:**
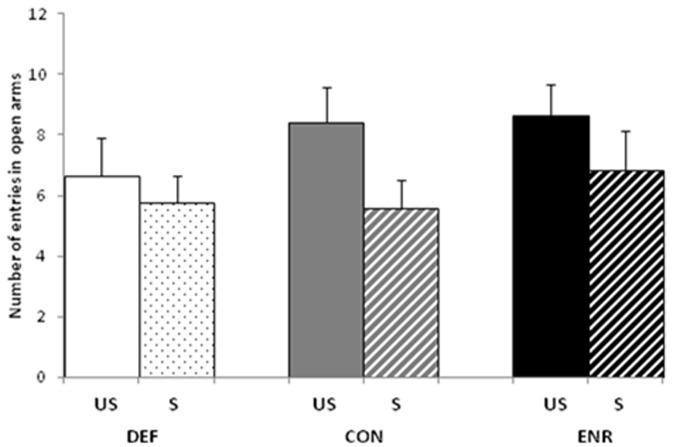
Performance in the Elevated Plus Maze (EPM). Mean number of entries into the open arms. Values are means ± SEM (n = 16 per group). US, unstressed; S, stressed; DEF, Deficient diet; CON, Control diet; ENR, Enriched diet.

The CON rats spent less than 10% of their time in the open arms under our conditions, which is less than the 20–30% reported in other studies [38]. We therefore could show no increase in anxiety (i.e. a decrease in the time spent in open arms) with this parameter. We therefore focused on the number of entries into the open and closed arms. Stress significantly decreased the number of entries into the open arms (Stress: F(1, 87) = 4.916, p<0.05), without any difference between the dietary groups (**Figure 4**). Neither diet nor stress significantly modified the percentage of entries into open arms (data not shown), suggesting that stress has a specific effect on locomotor activity, in agreement with the results obtained in the OF test.

#### Sucrose preference test

The percentages of sucrose preference between the stressed and unstressed rats in all three dietary groups were not significantly different (Diet: F(1, 85)<1; Stress: F(2, 85)<1): DEF group (unstressed: 63.8±5.2%; stressed: 63.2±5.7%), CON group (unstressed: 67.0±4.9%; stressed: 63.3±6.6%), ENR group (unstressed: 69.6±5.5%; stressed: 67.5±5.4%).

#### Startle response test

**Figure 5:**
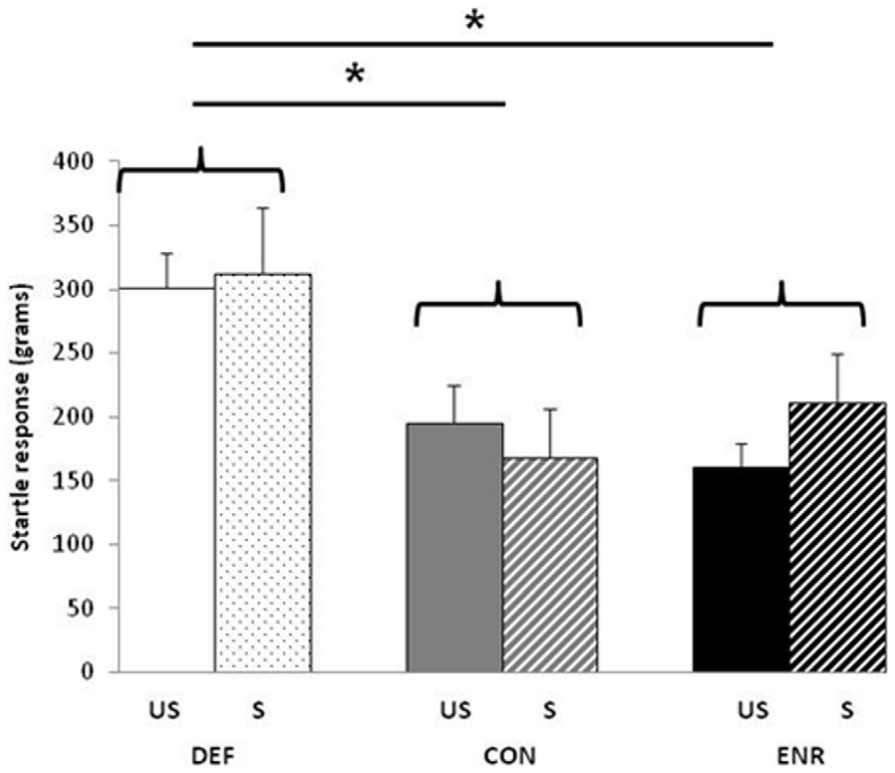
Startle response. Values are means ± SEM (grams; n = 16 per group). * significantly different between dietary conditions (ANOVA followed by MCP, *p<0.01*). US, unstressed; S, stressed; DEF, Deficient diet; CON, Control diet; ENR, Enriched diet.

The startle responses obtained for each of the groups are shown in **Figure 5**. The two-way ANOVA indicated a significant diet effect (F(2,81) = 7.594; p<0.01) and MCP revealed that the DEF rats had a higher startle response than ENR rats (p = 0.005) and than CON rats (p = 0.003). There was no effect of stress (F(1,81)<1).

### Lipid analyses

#### Cortex FA composition of PE

The main changes in the FA contents of the PE in frontal cortex (reproducing changes in the total brain) induced by the three diets and by chronic restraint stress concerned the omega-3 and the omega-6 FA, which influenced the overall total omega-6+omega-3 FA (**Tables 3**). The DEF rats had lower DHA than the CON rats, while this FA was higher in the ENR rats than in the CON rats (Diet: F(2,35) = 146.182; p<0.001). Conversely, the concentration of AA, a reflection of total omega-6 FA, in the DEF rats was higher than in the CON rats, and it was lower in the ENR rats than in the CON rats (Diet: F(2,35) = 57,875; p<0.001). Moreover, stress reduced the amounts of DHA in the CON (p = 0.038) and ENR rats (p = 0.009) (Stress: F(1,35) = 5,068; p<0.05), while it had no effect on the omega-6 FA. Although the total amounts of omega-6+omega-3 FA were equivalent in the unstressed rats in the three dietary groups, stress reduced these totals by 5% in the CON rats (p = 0.039) and ENR rats (p = 0.016) (Stress: (F(1,36) = 7.813; p<0.01). The overall proportions of saturated and monounsaturated FA were not substantially altered by either the diet or the stress.

**Table 3:** Main FA composition of phosphatidylethanolamine in the frontal cortex of unstressed or stressed rats fed the CON, ENR or DEF diets^1^^,^ 2.

	CON		ENR		DEF	
	unstressed	stressed	unstressed	stressed	unstressed	stressed
**Σ Saturated**	22.7±0.4	22.8±0.7	21.8±0.2	21.2±0.2	22.3±0.2	22.1±0.4
**Σ Monounsaturated**	12.7±0.6	13.7±0.6	14.0±0.2	15.6±0.3	13.3±0.3	13.4±0.3
**20:4 n-6**	12.6±0.2^a^	12.2±0.2	10.7±0.2^b^	10.2±0.2	13.6±0.2^c^	13.9±0.3
**22:5 n-6**	0.5±0.0^a^	0.5±0.0	0.2±0.0^a^	0.2±0.0	10.1±0.2^b^	9.9±0.2
**Σ omega-6 FA**	18.3±0.2^a^	18.0±0.2	14.9±0.2^b^	14.6±0.3	29.6±0.3^c^	29.9±0.5
**20:5 n-3**	0.1±0.0	0.1±0.0	0.2±0.0	0.1±0.0	0.1±0.0	0.1±0.1
**22:6 n-3**	25.1±0.7^a^	23.7±0.2	27.3±0.2^b^	25.6±0.5	12.9±0.4^c^	12.7±0.3
**Σ omega-3 FA**	25.7±0.8^a^	24.2±0.2	28.1±0.2^b^	26.3±0.5	13.2±0.4^c^	13.1±0.3
**Σ omega-6+omega-3 FA**	44.1±0.9	42.1±0.4	43.0±0.3	40.9±0.6	42.9±0.4	43.0±0.6

For each line, values with different letters (a–c) showed significant differences between the three dietary groups in the unstressed condition (ANOVA followed by MCP, *p<0.01*).

1 Values are means ± SEM (g/100 g TFA; n = 6 per group).

2 FA, fatty acids; 20:4n-6: arachidonic acid (AA); 22:5n-6: docosapentaenoic acid (DPA); 22:6n-3: docosahexaenoic acid (DHA); DEF, Deficient diet; CON, Control diet; ENR, Enriched diet.

#### Plasma FA composition (Table 4)

**Table 4:** Main plasma FA in total lipids of unstressed and stressed rats fed the CON, ENR or DEF diets^1^^,^ 2.

	CON		ENR		DEF	
	unstressed	stressed	unstressed	stressed	unstressed	stressed
**Σ Saturated**	29.8±0.3	29.9±0.6	31.9±0.7	33.2±0.7	30.4±0.4	31.1±0.3
**Σ Monounsaturated**	35.2±3.0^a^	31.8±2.7	30.4±1.1^b^	26.4±1.8	34.0±0.6^ab^	37.5±1.2
**20:4 n-6**	22.2±1.8^a^	21.6±2.7	11.0±0.5^b^	12.8±1.4	22.4±0.8^a^	21.6±1.0
**22:5 n-6**	0.1±0.0^a^	0.1±0.0	0.1±0.0^a^	0.1±0.0	2.6±0.2^b^	1.7±0.2
**Σ omega-6 FA**	32.4±1.6^a^	32.0±2.3	23.3±0.6^b^	26.4±0.8	34.0±0.7^a^	30.8±0.9
**20:5 n-3**	0.8±0.1^a^	0.8±0.1	5.3±0.3^b^	5.3±0.5	0.0±0.0^c^	0.0±0.1
**22:6 n-3**	3.2±0.3^a^	3.5±0.3	7.5±0.3^b^	7.3±0.3	0.4±0.0^c^	0.4±0.0
**Σ omega-3 FA**	5.1±0.2^a^	5.2±0.3	13.6±0.5^b^	13.3±0.6	0.5±0.0^c^	0.4±0.0
**Σ omega-6+omega-3 FA**	37.7±1.6	35.9±2.7	36.4±1.7	40.7±1.4	35.3±1.1	33.3±1.8

For each line, values with different letters (a–c) showed significant differences between the three dietary groups in the unstressed condition (ANOVA followed by MCP, *p<0.01*).

1 Values are means ± SEM (g/100 g of total fatty acids; n = 6 per group).

2 FA, fatty acids; 20:4n-6: arachidonic acid (AA); 22:5n-6: docosapentaenoic acid (DPA); 22:6n-3: docosahexaenoic acid (DHA); DEF, Deficient diet; CON, Control diet; ENR, Enriched diet.

The concentrations of FA in the plasma total lipids reflect the omega-3 FA in the diet and are similar to the diet-dependent profiles in the PE brain membranes. Thus, the special diets significantly altered the amounts of omega-3 and omega-6 FA (Diet: F(1,24) = 9.648 and 274.067 for omega-3 and omega-6, respectively; p<0.01) but stress induced no changes (F(1,24)<1). Then omega-3 levels were higher in plasma from rats under the ENR diet than those under the CON diet. The increase was mainly in DHA (p<0.001), but also 20:5n-3 (p<0.001), which is not incorporated into brain membranes. There were parallel decreases in the omega-6 FA, essentially because of a fall in AA (p<0.05). In contrast, the DEF diet produced a 10-fold decrease in omega-3 FAs (p<0.001) but no significant change in the total omega-6 FAs. The concentrations of saturated and monounsaturated FAs were not modified by diet or stress.

## Discussion

We have compared the stress response of adult rats fed diets containing different amounts of omega-3 FAs from conception, by exploring a wide range of emotional and biochemical stress parameters. The three diets used were either omega-3 FA-deficient, mimicking the poor omega-3 FA status of many western populations, contained a balance of omega-3 FA, as recommended by nutritional guidelines, or were supplemented with long chain omega-3 FA, to mimic the high DHA intakes associated with eating fish. Lipid analyses showed that the DHA concentration increased with the amount of omega-3 FA in the diet. Thus, the amounts of omega-3 FA in the plasma and PE of the frontal cortex were higher in the ENR rats than in the CON or DEF rats. This increase occurred at the expense of AA levels, which was decreased in both plasma and brain membranes. The incorporation of DHA into brain membrane phospholipids was especially effective when the diet contained DHA (as in the ENR diet) rather than its precursor (as in the CON diet), as previously described [46]. However, the chronic restraint stress procedure reduced the incorporation of DHA into brain membrane phospholipids, particularly in rats fed the CON and the ENR diets. We have previously shown that stress alters the phospholipid contents of brain membranes. Unpredictable chronic mild stress prevented the incorporation of DHA into the brain membranes of mice fed an omega-3-enriched diet [47], and the stress of maternal separation reduced the amounts of DHA incorporated into phospholipid membranes [18]. This suggests that stress influences the brain FA status, but the mechanism remains to be elucidated. An increase in lipid peroxidation has been observed in the cerebral tissues of chronically stressed rats, and this could contribute to the decreased DHA in the phospholipid membranes [9], [48], [49]. Chronic stress has also been found to have a persistent effect on circulating FA concentrations. Thus rats separated from their mothers and titi monkeys subjected to chronic social stress both had increased plasma omega-6/omega-3 FA ratios, suggesting a lower omega-3 FA status [50], [51]. However, the reduction in the amounts of DHA we observed in the PE of cortical membranes after the chronic restraint stress was not detected in the plasma. This suggests that the decrease in brain DHA is not due to a lack of plasma DHA. It could be linked to local dysregulation, such as changes in DHA incorporation into brain membranes, or its release or oxidation.

### Stress responses in rats on the CON diet

The 21 days of the stress procedure resulted in a gradual weight loss, as previously described [52], [53], in parallel with the reduced time of access to food. Nevertheless, all the experimental groups had the same body weight when they began the behavioral tests. Restraint stress was also associated with a rise in plasma corticosterone 30 min after the beginning of the constraint, which persisted until the restraint ended and reflected the activation of HPA [54], [55]. However, this increase in corticosterone was not associated with any change in the amounts of GR mRNA or protein in the hippocampus, measured one week after the end of the restraint. This suggests that the HPA axis was only moderately activated and did not durably alter the corticosterone pathway. Published data on the effects of chronic stress on GR expression in the hippocampus are contradictory. Some studies found that GR were down-regulated [25], [26], [56], while others observed no decrease in GR [57], [58]. Thus, the concentration of GR does not seem to be an immutable marker of chronic stress [57]–[59]. It has also been shown that the changes observed in hippocampal GR after 4 weeks of chronic stress are attenuated after recovery of 10 days [60]. Therefore, our finding of no change in hippocampal GR one week after the end of restraint stress, when the corticosterone peak had fallen, may also reflect a rapid recovery from the impact of stress. This apparent lack of effect on GR expression after high glucocorticoid secretion driven by chronic stress could be due to the dynamic regulation of GR and a return to baseline which is critical for maintaining homeostasis [61]. It seems now important to measure the GR expression immediately at the end of the stress procedure.

We evaluated the behavioural stress response by measuring locomotor activity and anxiety indices in the OF and the EPM tests. Spontaneous OF activity includes a variety of responses that can be interpreted as indices of exploration, arousal, locomotion, anxiety, and emotionality. In particular, increased locomotion (horizontal) and rearing (vertical) characterize the response of rats to novelty. We found that stress slightly increased the total horizontal and vertical activity, suggesting increased reactivity to novelty. There was a stress-induced decrease in the number of entries into the open arms of the EPM, together with a dramatic increase in grooming in the OF, suggesting increased anxiety. Grooming is an important component of the rodent behavioural repertoire and plays a major role in coping with stress [35]. Several studies have shown that stress increases the amount of grooming [36], [37]. By contrast, the measures of sucrose preference and the startle response, which are classically described as sensitive to chronic stress [32], [62], were not different in stressed and unstressed rats. This indicates that at the end-point of behavioural experiments, the rats previously subjected to the 21 days of restraint stress showed no signs of anhedonia or altered emotional reactivity. Thus, our study of the emotional behaviour of 6-month-old rats subjected to chronic restraint stress and fed a balanced omega-3 FA diet revealed only subtle differences from unstressed rats.

There are several experimental models of chronic stress, based either on physical and/or psychological constraints. Probably the most popular experimental way of inducing chronic stress in rodents is to use restraint, largely because it is painless [63], [64]. However, restraint can be applied in a number of ways, which makes comparison of studies difficult. Previous studies of emotional behaviour after repeated chronic stress have revealed mixed behavioural results. Some reported increased emotional behaviour [31]–[34], while others found no change [65], [66]. Furthermore, restraint will not necessarily produce a stress response, principally because the animal may adapt and become used to restraint. We postulate that the absence of any difference between the sucrose preference and startle responses between the stressed and unstressed CON rats reflects the habituation of rats to the restraint stress after 21 days. Overall, the 21-day restraint induced a measurable physiological stress response, with weight loss and a transient increase in corticosterone, and stress-related behavior, such as fewer entries into the open arms and increased grooming. However, the impact of the stress was moderate because it did not durably alter the HPA axis and did not produce marked signs of anxiety or depression.

We hypothesized that this moderate impact of restraint stress can be modulated by the brain omega-3 FA status. Numerous animal and clinical studies have investigated the effects of omega-3 FA dietary supplements on behaviour and have found that DHA may have an anti-stress function [17], [20], [67]. It has been shown that omega-3 FA deficient mice are more vulnerable to the stress of social isolation [16], and bright light and loud noise [14], in that they become more anxious than mice fed an omega-3 FA-adequate diet. We recently showed that rats fed an omega-3 FA deficient diet for two generations and subjected to the stress of early maternal separation were more anxious and fearful in inescapable situations, while their ability to cope with an aversive avoidance task remained unaffected [19]. This is why we analysed the responses to stress of rats fed an omega-3 FA DEF diet, or an omega-3 LC-FA ENR diet, associated with changes in FA brain membrane compositions, and compared these data with those for rats fed the CON diet balanced in omega-3 and omega-6 FAs.

### The omega-3 dietary supply impacts the stress response

Our results indicate that the stress response was attenuated by the chronic dietary enrichment of omega-3 FA, whereas the deficiency aggravated it. The 21-day restraint stress caused a sustained reduction in body weight, which did not occur in the unstressed rats, but the ENR rats lost less weight than did the CON and DEF groups, despite having the same food intake as the two other groups, suggesting a difference in metabolic rate. It has been shown that a reduced food intake and a loss of body weight after restraint stress are not clearly associated, and that many parameters such as NPY and monoamines contribute to this weight loss [53]. Better habituation to stress may explain this specific effect on the body weight of the ENR rats, as suggested by the lower corticosterone peak after 7 days of restraint stress than in the two other dietary groups. Ferraz et al. [21] recently described such a reduction in the stress-related hormone levels promoted by omega-3 FA supplementation, suggesting decreased activation of the HPA axis. Behavioural measures revealed that the ENR diet abolished the stress-induced reduction in locomotor activity in the OF. But the behavioural stress response of the rats on the DEF diet was greater than that of the two other dietary conditions, particularly when compared to ENR rats. The indices of anxiety, such as grooming and number of entries into open arms of the EPM, indicated that the restraint stress induced increased responses in all three dietary groups, but this response was much greater in the DEF rats, particularly for grooming. This suggests that DEF rats subjected to the chronic restraint stress were more anxious that the stressed rats of the two other dietary groups. Others have described an increased risk of anxiety-like behavior in rats on a diet deficient in omega-3 FA [68], whereas adequate levels of DHA or an increased supply lower anxiety [20]–[21]. Both stressed and unstressed DEF rats also exhibited an exaggerated startle response to an acoustic stimulus. Startle is a fast reflexive response to a sudden, intense stimulus, suggesting that it protects the individual against injury. It is a valuable tool for assessing the mechanisms of sensorimotor and emotional response plasticity whose magnitude can be influenced by a variety of pathological and experimental conditions [69]. Fedorova et al. [70] showed that a lack of omega-3 FA leads to a pronounced deficit in prepulse inhibition of the acoustic startle response, corresponding to a reduced startle response that is elicited when a distinctive non-startling acoustic stimulus is presented immediately before the startling stimulus. However, the authors did not report the first startle response, making it impossible to compare their findings with our results. Taken together, these results show that an omega-3 FA deficit increases emotional reactivity, as assessed by the startle response, but its neurobiological basis remains unclear.

We conclude that the dietary omega-3 FA supply modulates the responses of adult rats to chronic restraint stress. Although the diets used here were designed principally to influence the brain DHA content, this parameter, alone, does not predict the behavioral outcome. Thus a diet enriched in omega-3 FA attenuates some of the deleterious effects of chronic stress, particularly weight loss and the sustained corticosterone peak, whereas a lack of dietary omega-3 FA amplifies the behavioural indices of anxiety in stressed rats. This suggests that the omega-3 FA status influences sensitivity to stress, with an adequate supply of omega-3 FA providing better resilience and an inadequate supply resulting in a worse response.
